# Prevalence of *Giardia duodenalis* and *Cryptosporidium* species infections among children and cattle in North Shewa Zone, Ethiopia

**DOI:** 10.1186/1471-2334-13-419

**Published:** 2013-09-08

**Authors:** Teklu Wegayehu, Haileeyesus Adamu, Beyene Petros

**Affiliations:** 1 Department of Biology, College of Natural Sciences, Arba Minch University, Arba Minch, Ethiopia; 2 Aklilu Lemma Institute of Pathobiology, Addis Ababa University, Addis Ababa, Ethiopia; 3 Centers for Disease Control and Prevention (CDC), 1600 Clifton Rd, Atlanta, GA 30329, USA; 4 Department of Microbial, Cellular and Molecular Biology, College of Natural Sciences, Addis Ababa University, Addis Ababa, Ethiopia

**Keywords:** *Giardia duodenalis*, *Cryptosporidium*, Prevalence, Zoonotic transmission

## Abstract

**Background:**

*Giardia* and *Cryptosporidium* are the most common causes of protozoan diarrhea that lead to significant morbidity and mortality worldwide. The purpose of this study was to determine the prevalence of *Giardia duodenalis* and *Cryptosporidium* species infections among children and cattle, and to assess the potential risk of zoonotic transmission.

**Methods:**

This cross-sectional study was conducted between January and April 2009 in Girar Jarso and Dera Districts of North Shewa Zone, Oromia Region, Ethiopia. A total of 768 stool specimens were collected and examined for intestinal parasites using direct wet mount with saline and formalin ether concentration methods. The modified Ziehl-Neelsen staining method was used for the detection of *Cryptosporidium* species. Statistical analysis was performed using SPSS software version 15.

**Results:**

Out of 384 children examined, 53 (13.8%) and 28 (7.3%) were positive for *Giardia* and *Cryptosporidium* infections, respectively. Similarly, of the total 384 cattle examined, 9 (2.3%) were positive for *Giardia duodenalis* and 30 (7.8%) were positive for *Cryptosporidium* infection. The prevalence of giardiasis was significantly higher among children who had close contact with cattle 33 (18.7%) compared to children who had no contact with cattle 20 (9.6%) (P < 0.05). Higher number of *Cryptosporidium* infection was also recorded in children who had close contact with cattle 15 (8.5%). Difference in prevalence of giardiasis and cryptosporidiosis among children was not statistically significant between males and females. On the other hand, difference in the prevalence of giardiasis among children was statistically significant between age groups.

**Conclusions:**

Higher prevalence of *Giardia duodenalis* infection detected among children was significantly associated with contact with cattle and manure that the children had. Further analysis using molecular techniques is needed to explain the existence of zoonotic transmission in the study area.

## Background

*Giardia* and *Cryptosporidium* are the most common causes of protozoan diarrhea that lead to significant morbidity and mortality in the developing and developed world. They are transmitted through the fecal-oral route following direct or indirect contact with the infective stages of the parasite from three sources: anthroponotic, zoonotic and sapronotic [1,2]. *Giardia* and *Cryptosporidium* infections are common cause of gastroenteritis known as giardiasis and cryptosporidiosis, respectively.

Both *Giardia* and *Cryptosporidium* share a broad host range and are believed to be zoonosis [3]. Despite our knowledge of the distribution of these species among more than hundred mammalian species and numerous reports from human communities, the routes of environmental transmission are still not well defined [4,5]. This is attributed to the fact that each genus is believed to comprise complex of species and genotypes within the species, some of which are pathogenic, others specific to particular hosts and some zoonotic, and hence of public health significance [5,6].

Epidemiological surveys indicated that the most important sources of human infection are contaminated drinking and recreational water, food, household animals and infected people [7]. Sources of contamination of water and food might be diverse, but a particularly important, albeit varying, role is played by different host groups that act as reservoirs of infection. Farm animals are believed to play the most significant role in this context, contributing parasite cysts/oocysts in large proportion because of their high abundance on farms [7].

Farmers in Ethiopia are engaged in mixed agricultural practice and use cow manure as a fertilizer and dried dung as fuel. In such localities where people have close contact with animals and their manure, the possibility of infection with zoonotic pathogens such as *Giardia duodenalis* (*G. duodenalis*) and *Cryptosporidium* species is high. Although a number of studies have been conducted on the distribution and prevalence of *G. duodenalis* and *Cryptosporidium* species in different parts of Ethiopia [8-15], none of these previous works had determined the prevalence in human and cattle located in the same region and spanning the same time period. Therefore, the objective of the present study was to determine the prevalence of *G. duodenalis* and *Cryptosporidium* species infections in children and cattle, and to assess the risk of zoonotic transmission.

## Methods

### Study area

**Figure 1:**
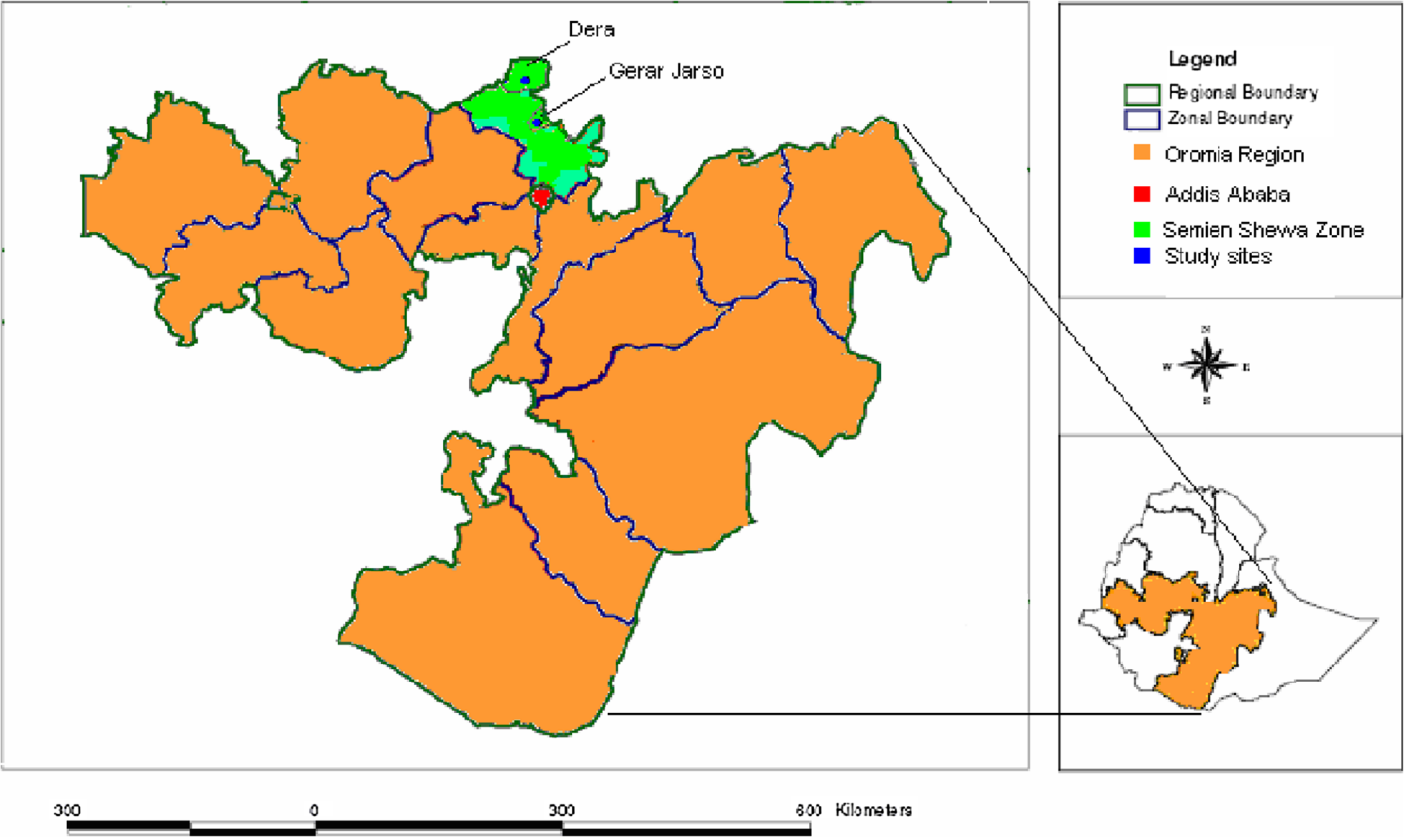
**Map of Ethiopia showing the location of study sites, Girar Jarso and Dera.** The orange and green colors indicate the Oromia Regional State and North Shewa Zone, respectively. The blue color designates Girar Jarso and Dera districts. The red color shows the capital of Ethiopia, Addis Ababa.

This cross-sectional study was conducted between January and April 2009 in Girar Jarso and Dera districts of North Shewa Zone, Oromia Region, Ethiopia (Figure 1). The zone is one of the 18 zones in the region and is the second nearest zone to Addis Ababa. Mixed farming is the major livelihood of the people and the livestock owned mainly includes cattle, sheep, goat, equine and poultry. The expansion of social services, secondary economic activities and modern means of transportation and communication in the zone are similar to other zones of the region. Factors possibly causing differences in prevalence of intestinal parasites such as source of drinking water, level of education, the presence of a latrine and other social and environmental factors are comparable in the study area.

### Sample size and sampling techniques

The sample size was determined using the single proportion population formula. It was calculated assuming a prevalence of 50% with a margin of error of 0.05 and a confidence level of 95%. In line with it, 384 households for the sample were calculated and of which a total of 768 unit of analysis, 384 children of ages between 1 to 14 years and 384 cattle of different age groups, were selected. About 378 subjects (187 children and 191 cattle) were sampled from Girar Jarso district and 390 subjects (197 children and 193 cattle) were sampled from Dera district. Out of 384 children examined, 176 have cattle at their home and vicinity and hence close contact with cattle. The remaining 208 children do not have cattle at their home and vicinity i.e. had no contact with cattle and their manure. Multistage sampling technique was employed for the purpose of sampling. Two districts from the zone and one kebele from each district were selected randomly. The first household was randomly selected in each kebele and the consecutive households were designated using systematic sampling method. Finally, the children and cattle were randomly selected from each household.

### Fecal sample collection and processing

A single fresh fecal sample was collected from each consenting study subject in a labeled and sterile fecal container. The questionnaires concerning socio-demographic characteristics of the study participants (age and sex), contact with animals and their manure, and the tag number of the cattle were filled by all the study participants’ parents or guardians during the sample collection. A portion of stool was examined at field by direct wet mount with saline (0.85% sodium chloride solution) to observe motile intestinal parasites and trophozoites under light microscope at 10X and 40X magnifications. Lugol’s iodine staining technique was also done to observe cysts of the intestinal protozoan parasites. The remaining part was preserved with 10% formalin in the ratio of 1 gram of stool to 3 ml of formalin and processed by the following methods.

### Formalin ether concentration

About 7 ml of 10% formalin was added to approximately 1 gram of feces and mixed using an applicator stick. The stool sample was sieved with cotton gauze and transferred to 15 ml centrifuge tube. After adding 3 ml of diethyl ether to the mixture and hand shaken, the content was centrifuged at 2000 rpm for 3 minutes. The supernatant was poured way and a drop of sediment was transferred to slide. Finally, the entire area under the cover slip was systematically examined using 10X and 40X objective lenses to observe ova, cyst and larvae of different intestinal parasites [16].

### Modified Ziehl-Neelsen technique

In this method thin smears were prepared from preserved as well as sediments of concentrated stool samples, air-dried, and fixed with absolute methanol for 5 minutes. The smears were stained with carbol-fuchsin for 30 minutes and thereafter, washed with tap water. The slides were decolorized in acid alcohol for 2 minutes and were counter stained with methlyene blue for another 2 minutes. Finally the stained smears were examined using oil immersion objective to detect oocysts of *Cryptosporidium*[17].

### Quality control

Before starting the actual work, quality of reagents and instruments were checked by experienced laboratory technologist. The specimens were also checked for serial number, quality and procedures of collection. Each stool sample was examined by two laboratory technicians. The laboratory technicians were not informed about the health and other status of the study participants to eliminate observer bias. In cases where the results were discordant, a third senior technician was used, and his report was considered the final result. Randomly selected samples were also sent to other laboratories and checked for the reproducibility of the results. All data were entered timely to the database and checked for its accuracy before proceeding to analysis.

### Data analysis

Statistical analysis was performed with SPSS software version 15. Chi square test was used to verify possible association between *G. duodenalis* and *Cryptosporidium* species infections, and exposure to different factors. Values were considered to be statistically significant when the calculated P-value was less than 0.05.

### Ethics statement

The study was reviewed and approved by the Ethical Review Committee of Biology Department, Addis Ababa University. The health care managers of each district health centre and the administrative authorities at community level were informed about the study and their consent was obtained. Parents/guardians of participating children signed a written informed consent prior to study enrolment. Positive individuals were treated with standard drugs, which were administered by the site physicians.

## Results

A total of 768 fecal samples were collected from 384 children and 384 cattle. Children were stratified in two age groups: 57 (14.8%) children under age group 1-5 years and 327 (85.2%) children under age group 6-14 years. The study included 191 (49.7%) males and 193 (50.3%) females with male to female ratio of 1:1.01. The overall prevalence of *Giardia* and *Cryptosporidium* infections in children was 53 (13.8%) and 28 (7.3%), respectively. Likewise, the overall prevalence of these parasites in cattle was 9 (2.3%) and 30 (7.8%), in the preceding order (Table 1).

**Table 1:** **Prevalence of *G. duodenalis *and *Cryptosporidium *species among children and cattle in the study sites**

**Study subjects**	**Study sites**	**Number of subjects examined**	**Parasites identified**					
			***G. duodenalis***			***Cryptosporidium *species**		
			**No (%)**	***χ***^**2**^	**P-value**	**No (%)**	***χ***^**2**^	**P-value**
**Children**	Girar Jarso	187	33 (17.6)			15 (8.0)		
	Dera	197	20 (10.1)	**4.53**	0.03^*^	13 (6.6)	**0.28**	**0.59**
	**Total**	**384**	**53 (13.8)**			**28 (7.3)**		
**Cattle**	Girar Jarso	191	6 (3.1)			23 (12.0)		
	Dera	193	3 (1.5)	**1.05**	**0.30**	7 (3.6)	**9.43**	0.00^*^
	**Total**	**384**	**9 (2.3)**			**30 (7.8)**		

**Key:***χ*2 and P- values compare the prevalence between Girar Jarso and Dera in children and cattle.

* Represent statistically significant difference (P < 0.05).

### *Giardia* and *Cryptosporidium* infections among children and cattle

In Girar Jarso district, 187 stool samples were examined from children. Out of these 33 (17.6%) were positive for *Giardia* and 15 (8.0%) were positive for *Cryptosporidium* infections. In Dera district, out of 197 samples examined 20 (10.1%) and 13 (6.6%) were positive for *Giardia* and *Cryptosporidium*, respectively. The prevalence of giardiasis showed significant difference (P < 0.05) between Girar Jarso and Dera district but non-significant for cryptosporidiosis (Table 1).

Out of 191 fecal samples collected from cattle in Girar Jarso, 6 (3.1%) and 23 (12.0%) were positive for *Giardia* and *Cryptosporidium* infections, successively. In the same expression, out of 193 fecal specimens collected and examined from cattle in Dera, 3 (1.5%) were positive for *Giardia* and 7 (3.6%) were positive for *Cryptosporidium* infections. The prevalence of cryptosporidiosis in cattle showed a significant difference (P < 0.05) between the study sites (Table 1).

### *Giardia* and *Cryptosporidium* infections among children with respect to contact with cattle

To see the distribution of *G. duodenalis* and *Cryptosporidium* species infections among children in relation to contact with cattle and their manure, data were arranged and summarized in Table 2. Out of 176 stool samples collected from children who had close contact with cattle and their manure, 33 (18.7%) were positive for *Giardia* and 15 (8.5%) were positive for *Cryptosporidium*. Among the 208 study participants who had no contact with cattle, 20 (9.6%) and 13 (6.2%) were positive for *Giardia* and *Cryptosporidium* infections, respectively (Table 2). The analysis of the prevalence of *Giardia* infection was significantly higher (P < 0.05) among children who had close contact with cattle compared to those who had no contact. Higher number of *Cryptosporidium* infection was observed in children who had close contact with cattle 15 (8.5%) than those who had not contact 13 (6.2%).

**Table 2:** **Prevalence of *G. duodenalis *and *Cryptosporidium *species among children with respect to contact with cattle**

**Contact with cattle**	**Number of subjects examined**	**Parasites identified**					
		***G. duodenalis***			***Cryptosporidium *species**		
		**No (%)**	***χ***^**2**^	**P-value**	**No (%)**	***χ***^**2**^	**P-value**
Contact	176	33 (18.7)			15 (8.5)		
No contact	208	20 (9.6)	**9.43**	0.00^*^	13 (6.2)	**0.73**	**0.39**
**Total**	**384**	**53 (13.8)**			**28 (7.3)**		

**Key:***χ*2 and P- values compare the prevalence with respect to contact with cattle.

* Represent statistically significant difference (P < 0.05).

### *Giardia* and *Cryptosporidium* infections among children by sex and age groups

Out of the 384 children, 191 were males and 193 were females. The prevalence of giardiasis was 27 (14.1%) in males and 26 (13.4%) in females. Similarly, the prevalence of cryptosporidiosis was 15 (7.8%) and 13 (6.7%) in males and females, respectively. The difference in the prevalence of *Giardia* and *Cryptosporidium* infections was not statistically significant between males and females (Table 3).

**Table 3:** **Prevalence of *G. duodenalis *and *Cryptosporidium *species among children by sex and age groups**

**Demographic profile**	**Number of children examined**	**Parasites identified**					
		***G. duodenalis***			***Cryptosporidium *species**		
		**No (%)**	***χ***^**2**^	**P-value**	**No. (%)**	***χ***^**2**^	**P-value**
**Sex**							
Male	191	27 (14.1)	**0.03**	**0.85**	15 (7.8)	**0.17**	**0.67**
Female	193	26 (13.4)			13 (6.7)		
**Age Group**							
1-5	57	14 (24.5)	**6.51**	0.01^*^	7 (12.3)	**2.46**	**0.11**
6-14	327	39 (11.9)			21 (6.4)		

**Key:***χ*2 and P- values compare the prevalence between male and female and between age groups.

* Represent statistically significant difference (P < 0.05).

Regarding age groups, out of the 384 children, 57 were 1 to 5 years and 327 were 6 to 14 years age category. Among the 57 children in the 1 to 5 years age category, 14 (24.5%) were positive for *Giardia* and 7 (12.3%) for *Cryptosporidium* infections. Of the 327 children of age between 6 to 14 years, 39 (11.9%) and 21 (6.4%) were positive for *Giardia* and *Cryptosporidium*, respectively. Difference in the prevalence of *Giardia* among children was statistically significant (P < 0.05) between age groups but non-significant for *Cryptosporidium* (Table 3).

### Intestinal parasites other than *G. duodenalis* and *Cryptosporidium*

Different types of intestinal parasites other than *G. duodenalis* and *Cryptosporidium* were identified in the study areas (Table 4). The prevalence of *Entamoeba histolytica/dispar* was the highest being 14 (3.6%), followed by *Hymenolepis nana* 11 (2.8%), *Ascaris lumbricoides* 7 (1.8%), *Enterobius vermicularis* 4 (1.0%)*,* and that of *Strongyloides stercoralis, Trichuris trichiura* and hookworm 2 (0.5%) each. Although single parasite infection had the highest prevalence, there was also double infection. Overall coinfection was detected in 10 (2.6%) of the study subjects. Among the double parasitic infection, *G. duodenalis* and *Cryptosporidium* comprised the highest proportion followed by *G. duodenalis*/*Trichuris trichiura* and *Hymenolopis nana*/*Strongyloides stercoralis*.

**Table 4:** Prevalence of other intestinal parasites among children in Girar Jarso and Dera sites

**Parasite identified**	**Study sites**		**Total (n = 384) No (%)**
	**Girar Jarso (n = 187) No (%)**	**Dera (n = 197) No (%)**	
*Entamoeba histolytica/dispar*	5 (2.6)	9 (4.5)	14 (3.6)
*Ascaris lumbricoides*	3 (1.6)	4 (2.0)	7 (1.8)
*Hymenolopis nana*	5 (2.6)	6 (3.0)	11 (2.8)
*Enterobius vermicularis*	1 (0.5)	3 (1.5)	4 (1.0)
*Strongyloides stercoralis*	-	2 (1.0)	2 (0.5)
*Trichuris trichiura*	-	2 (1.0)	2 (0.5)
Hookworm	1 (0.5)	1 (0.5)	2 (0.5)

## Discussion

The present study determines the prevalence of *G. duodenalis* and *Cryptosporidium* species infections among children and cattle in two randomly selected districts in North Shewa Zone. The prevalence of *Giardia* infection in children (13.8%) was not much different from (9.3%) reported by Seyoum *et al.*[14] from preschool children in Addis Ababa. On the other hand, the present finding was far lower than 35.3% prevalence reported by Ayalew *et al.* from children in Lege Dini, Ethiopia [12]. The possible explanations for the discrepancy between the present and previous findings might be the variation in sampling techniques used, the difference in the quality of drinking water source, and variation in the environmental condition of the study localities.

The prevalence of *Cryptosporidium* infection recorded in this study, where 7.3% of fecal samples were positive (Additional file 1), was lower than that of *Giardia* (13.8%). The prevalence was in agreement with the situation in northwestern Ethiopia as reported by Mersha and Tiruneh [8] where *Cryptosporidium* infection among children was 9%. The result was also comparable to the 12.2% prevalence reported from Lege Dini, Dire Dawa for children by Ayalew *et al.*[12]. Although the children had not shown signs and symptoms of the diseases, the prevalence was higher than the earlier reports from Addis Ababa and Jimma in symptomatic children with rates of 5.6% [9] and 3.3% [11], respectively. However, it was much lower than the 25.9% detected in AIDS patients with chronic diarrhea from Addis Ababa Hospitals [10]. A reason for differences in prevalence figures is probably because urban settings have better hygienic condition than rural settings and immune-deficiency is a risk factor for *Cryptosporidium* infection.

The zoonotic potential of giardiasis is under debate for many years. However, the high prevalence of infection in cattle and sheep together with reports that cattle and sheep can shed cysts of zoonotic types that infect humans [18,19] warrants further attention. The 2.3% of *Giardia* infection in cattle in this study is comparable to the 3.7% reported by Degerli *et al.*[20] in cows and calves from Turkey. Nevertheless, it was much lower than the 23% prevalence in healthy calves in Sweden [21].

*Cryptosporidium parvum* in livestock has been shown to be an important reservoir for cryptosporidiosis in humans, and contaminated water may be a key vehicle for the parasite [22]. It is therefore noteworthy that 7.8% of the cattle in the present study were oocyst shedding, which may account for environmental contamination of the study area. When compared to the few published sources overseas, the present finding is within the reported range of prevalence (0 to 40%) in adults and unspecified age group [23,24]. However, it was lower than 17. 6% reported by Abebe *et al.*[25] in dairy calves on selected dairy farms of central Ethiopia. The discrepancy could be due to age related variation, seeing that *Cryptosporidium* species infections are more prevalent in calves than adults [26].

*Giardia* and *Cryptosporidium* from cattle are potential zoonotic pathogens, and contact with their manure is believed the major risk factor for infection in humans [22]. In this study, children who had close contact with cattle and their manure have shown significantly higher prevalence of *G. duodenalis* infection when compared with those who had no contact with cattle (P = 0.00). The possible reason for these differences between the two study groups is that, in addition to anthroponotic transmission, there might be zoonotic transmission of the parasite from the infected cattle. Higher number of *Cryptosporidium* infection was also recorded in children who had close contact with cattle. This suggests the need for molecular techniques to identify genotypes and subtypes of the two parasites to clarify the sources and transmission pattern in the study area.

Although Salas [27] had reported *G. duodenalis* infection to be higher in males than in females among children 0 to 10 years of age, no such sex-associated prevalence was observed in the present study. This lack of difference in the prevalence rates of both *G. duodenalis* and *Cryptosporidium* species in children was in agreement with a study in Philippines where the gender of the children did not influence the rate of infection with these parasites [28]. The possible reasons for the absence of sex-related difference in the prevalence among the children could be explained by the observation that all children irrespective of their sex participate equally in the domestic chores that included cattle herding and manure handling in the study communities. Besides, the hygienic practices exercised by children of both sexes were also essentially similar.

A significantly high prevalence of giardiasis among children in the age groups 1-5 years in the study sites is in agreement with what was reported elsewhere [29,30]. Similar report by CDC [31] also showed age-specific incidence of giardiasis to be highest in children aged 1-4 years followed by children aged 5-14 years in Los Angeles. The possible justifications for the lower prevalence of infection occurring in the 6-14 years age group could be due to the incremental development of immunity, better awareness in washing hands and maintaining other personal hygienic measures with increasing age.

Although significant difference was not observed, *Cryptosporidium* species infection was slightly higher among 1-5 years age group than 6-14 years age group. This is in accordance with report of Casemore [32], who showed peak incidence of cryptosporidiosis to be among children aged 1-5 years. Similarly, in Manitoba, Canada, *Cryptosporidium parvum* was isolated much more frequently from fecal specimens collected from children under five years of age [33]. However, this finding is dissimilar to the report of Lindo *et al*. [16] in Jamaica, and others [17,34] from sub-Saharan African countries, including Uganda and Gambia, and Assefa *et al.*[9] from Ethiopia, where a difference in prevalence between age groups was observed.

In this work intestinal parasitic infection other than *G. duodenalis* and *Cryptosporidium* species were also identified among the children. Unlike reports from other localities, the prevalence of intestinal helminths was much less than what was reported from 10-33 years age [13,14,35] in Ethiopia. The low prevalence of helminths in the present study localities might be associated with the de-worming program launched by enhanced outreach strategy. The level of double infection with intestinal parasites determined in the present study (2.6%) was much lower than what was reported from southwest Ethiopia portraying a double infection of 35.8% among urban communities [15]. The difference in the socio-demographic condition and the involvement of all age groups in the previous study might explain the observed difference in double infection in the two study localities.

This study did not identify the genotypes and subtypes of the parasites due to lack of facilities in our laboratory. Nonetheless, the results are still important because there is little information available in the area. The age of the cattle we sampled was unspecified because of lack of information and this can be taken as one of the limitations since *Cryptosporidium* infection in cattle are age related. Apart from these limitations our study has the following strengths. It is first work to report the prevalence of the two parasites both in humans and cattle in the districts. In addition, standard laboratory techniques were used and all laboratory works were followed standard procedures.

## Conclusion

This study showed that *G. duodenalis* and *Cryptosporidium* species infections were widespread in varying magnitude among children and cattle in the study area. Significantly higher prevalence of *G. duodenalis* was detected among children who had close contact with cattle than those who had no contact. This suggests that contact with cattle and their manure could be a risk factor for infection with this protozoan parasite. Therefore, molecular techniques are necessary to clarify the source of infection and transmission pattern of the parasites. Moreover, measures including health education on personal and environmental hygiene would help in reducing the prevalence of these parasites.

## Competing interests

The authors declare that they have no competing interests.

## Authors’ contributions

TW conception of the research idea, designing, collection of data, data analysis, interpretation, and manuscript drafting. HA data analysis and manuscript reviewing. BP conception of the research idea, designing, data analysis, interpretation, and manuscript reviewing. All Authors read and approved the final manuscript.

## Pre-publication history

The pre-publication history for this paper can be accessed here:

http://www.biomedcentral.com/1471-2334/13/419/prepub

## Supplementary Material

Additional file 1 **Red stained oocysts of *Cryptosporidium *in modified Ziehl-Neelsen stained faecal preparation observed under microscope (magnification 1000x).** Click here for file

## References

[B1] Caccio SM Thompson RC McLauchlin J Smith HV Unravelling *Cryptosporidium* and *Giardia* epidemiology Trends Parasitol 2005 21 430 437 10.1016/j.pt.2005.06.013 16046184

[B2] Hubalek Z Emerging human infectious diseases: anthroponoses, zoonoses, and sapronoses Emer Infect Dis 2003 9 403 404 10.3201/eid0903.020208 PMC2958532 12643844

[B3] Monis PT Thompson RC *Cryptosporidium* and *Giardia* zoonoses: fact or fiction? Infect Genet Evol 2003 3 233 244 10.1016/j.meegid.2003.08.003 14636685

[B4] Appelbee AJ Thompson RC Olson ME *Giardia* and C*ryptosporidium* in mammalian wildlife-current status and future needs Trends Parasitol 2005 21 370 376 10.1016/j.pt.2005.06.004 15982929 PMC7185620

[B5] Smith HV Caccio SM Tait A McLauchlin J Thompson RC Tools for investigating the environmental transmission of *Cryptosporidium* and *Giardia* infections in humans Trends Parasitol 2006 22 160 167 10.1016/j.pt.2006.02.009 16503418

[B6] Caccio SM Thompson RC McLauchlin J Smith HW Unravelling *Cryptosporidium* and *Giardia* epidemiology Trends Parasitol 2005 21 431 437 10.1016/j.pt.2005.06.013 16046184

[B7] Dillingham RA Lima AA Guerrant RL Cryptosporidiosis: epidemiology and impact Microb Infect 2002 4 1059 1066 10.1016/S1286-4579(02)01630-1 12191656

[B8] Mersha D Tiruneh M Frequency of *Cryptosporidium* oocysts in Ethiopian children with diarrhoeal disease East Afr Med J 1992 69 314 315 1505416

[B9] Assefa T Mohammed H Abebe A Abebe S Tafesse B Cryptosporidiosis in children seen at the children’s clinic of Yekatit 12 hospital, Addis Ababa Ethiop Med J 1996 34 43 45 8674499

[B10] Fisseha B Petros B Woldemichael T *Cryptosporidium* and other parasites in Ethiopian AIDS patients with chronic diarrhoea East Afr Med J 1998 75 100 101 9640833

[B11] Gebru K Girma M Prevalence of *Cryptosporidium* infection in children at the pediatrics clinic of Jimma Hospital, Southwest Ethiopia Ethiop J Health Sci 2000 10 123 127

[B12] Ayalew D Boelee E Endeshaw T Petros B *Cryptosporidium* and *Giardia* infection and drinking water source among Children in Lege Dani, Ethiopia Trop Med Int Health 2008 13 4 472 475 10.1111/j.1365-3156.2008.02024.x 18282239

[B13] McConnel E Armstrong JC Intestinal parasitism in fifty communities on the central plateau of Ethiopia Ethiop Med J 1976 14 159 169 1026424

[B14] Seyoum T Abdulahi Y Haile-Meskel F Intestinal parasitic infection in preschool children in Addis Ababa Ethiop Med J 1981 19 35 40 7285890

[B15] Mengistu A Gebre-Selassie S Kassa T Prevalence of intestinal parasitic infections among urban dwellers in southwest Ethiopia Ethiop J Health Dev 2007 21 1 12 17

[B16] Lindo FJ Levy AV Baum KM Palmer JC Epidemiology of giardiasis and cryptosporidiosis in Jamaica Am J Trop Med Hyg 1998 59 5 717 721 9840587 10.4269/ajtmh.1998.59.717

[B17] Adegbola RA Demba E Deveer G Todd F *Cryptosporidium* infection in Gambian children less than 5 years of age Am J Trop Med Hyg 1994 97 103 107 8169999

[B18] O’Handley RM Olson ME Fraser D Adams P Thompson RC Prevalence and genotypic characterization of *Giardia* in dairy calves from Western Australia and Western Canada Vet Parasitol 2000 90 193 200 10.1016/S0304-4017(00)00235-1 10841999

[B19] Keulen H Macechko PT Wade S Schaaf S Wallis PM Erlandsen SL Presence of human *Giardia* in domestic, farm and wild animals, and environmental samples suggestes a zoonotic potential for giardiasis Vet Parasitol 2002 108 97 107 10.1016/S0304-4017(02)00181-4 12208038

[B20] Degerli S Celiksoz A Kalkan K Ozcelik S Prevalence of *Cryptosporidium* spp. and *Giardia* spp. in cows and calves in Sivas Turk J Vet Anim Sci 2005 29 995 999

[B21] Bjorkman C Svensson C Christensson B Verdier K *Cryptosporidium parvum* and *Giardia intestinalis* in calf diarrhea in Sweden Acta Ves Scand 2003 44 3 4 10.1186/1751-0147-44-145 PMC1831560 15074627

[B22] Olson ME O’Handley RM Ralston BJ McAllister TA Thompson RC Update on *Cryptosporidium* and *Giardia* infections in cattle Trends Parasitol 2004 20 185 10.1016/j.pt.2004.01.015 15099558

[B23] Anderson BC Abomasal cryptosporidiosis in cattle Vet Pathol 1987 24 235 238 3603962 10.1177/030098588702400307

[B24] Ralston BJ Mcallister TA Olson ME Prevalence and infection pattern of naturally acquired giardiasis and cryptosporidiosis in range beef calves and their dams Vet Parasitol 2003 114 113 122 10.1016/S0304-4017(03)00134-1 12781473

[B25] Abebe R Wossene A Kumsa B An epidemiological study of *Cryptosporidium* infection in dairy calves on selected dairy farms of central Ethiopia Rev Med Vet 2008 159 2 107 111

[B26] Graaf DC Vanopdenbosch E Ortega-Mora LM Abbassi H Peeters JE A review of the importance of cryptosporidiosis in farm animals Int J Parasitol 1999 29 1269 1287 10.1016/S0020-7519(99)00076-4 10576578 PMC7127282

[B27] Salas S Prevalence of giardiasis among patients at the University of San Agustin Clinical Laboratory Agustinian 1997 1 89 100

[B28] Natividad FF Buerano CC Lago CB Mapua CA de Guzman BB Seraspe EB Lorena P Samentar LP Endo T Prevalence rates of *Giardia* and *Cryptosporidium* among diarrheic patients in the Philippines Southeast Asian J Trop Med Public Health 2008 39 991 999 19062686

[B29] Isaac-Renton JL Philion JJ Factors associated with acquiring giardiasis in British Columbia residents Can J Public Health 1992 83 2 155 158 1617559

[B30] Harter L Frost P Jakubowski W *Giardia* prevalence among 1-to-3-year-old children in two Washington State counties Am J Public Health 1982 72 4 386 388 10.2105/AJPH.72.4.386 7065318 PMC1649908

[B31] Centers for Disease Control and Prevention (CDC) Giardiasis Surveillance United States, 1992-1997 MMWR 2000 Atlanta: CDC

[B32] Casemore DP Epidemiological aspects of human cryptosporidiosis Epidemiol Infect 1990 104 1 28 10.1017/S0950268800054480 2407541 PMC2271741

[B33] Mann ED Selka LH Nayar GP Koschik C Infection with *Cryptosporidium spp.* in humans and cattle in Manitoba Can J Vet Res 1986 50 174 178 3756670 PMC1255185

[B34] Tumwine JK Kekitiinwa A Nabukeera N Akiyoshi DE Rich SM Widmer G Feng X Tzipori S *Cryptosporidium parvum* in children with diarrhoea in Mulago Hospital, Kampala, Uganda Am J Trop Med Hyg 2003 68 710 715 12887032

[B35] Ali I Mekete G Wodajo N Intestinal parasitism and related risk factors among students of Asendabo Elementary and Junior Secondary school, South western Ethiopia Ethiop J Health Dev 1999 13 2 1 5

