# Supplementary figures and images for: Prevalence of Giardia duodenalis and Cryptosporidium species infections among children and cattle in North Shewa Zone, Ethiopia
Source: BMC Infect Dis. 2013 Sep 8;13:419. doi: 10.1186/1471-2334-13-419 (PMC3849630; doi:10.1186/1471-2334-13-419)

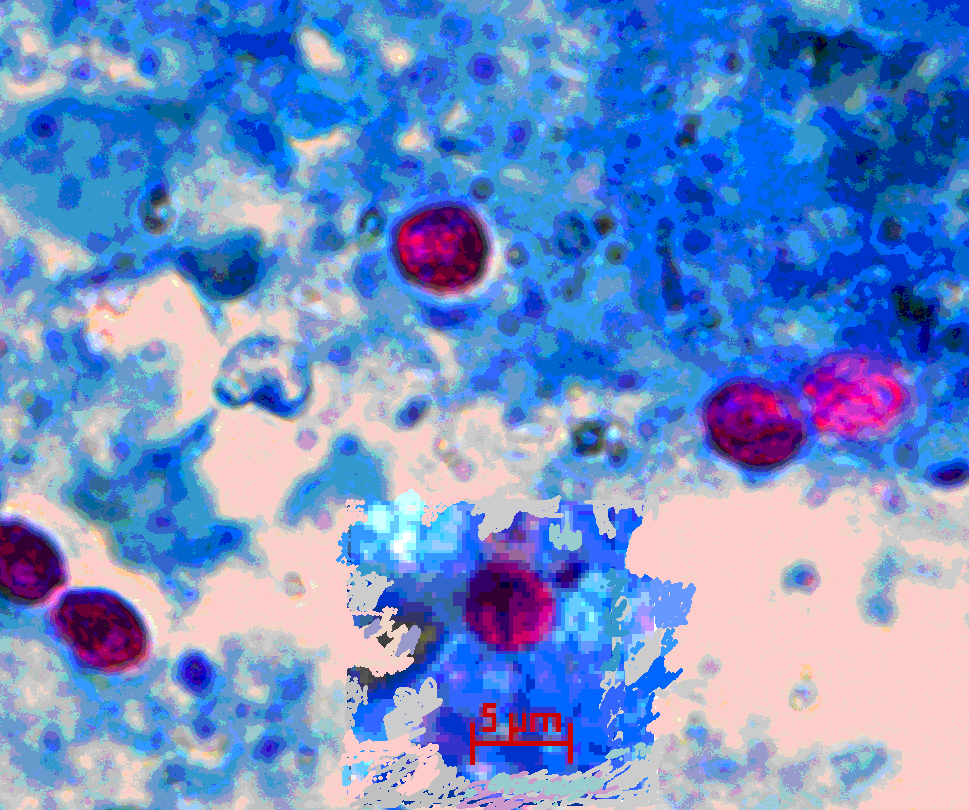

Supplement: Additional file 1 — Red stained oocysts of Cryptosporidium in modified Ziehl-Neelsen stained faecal preparation observed under microscope (magnification 1000x). [file 1471-2334-13-419-S1.docx]
